# “You’re a trainee telling your consultant to hold their question until later“: Using a resident-led faculty development workshop to explore trainee-consultant expertise role-reversal

**DOI:** 10.3205/zma001789

**Published:** 2025-11-17

**Authors:** Beatrice B. Preti, Claire P. Browne, Michael S. Sanatani, Christopher J. Watling

**Affiliations:** 1 Western University, Department of Oncology, London (ON), Canada; 2 Emory University, Department of Haematology & Medical Oncology, Atlanta (GA), USA; 3 Western University, Schulich School of Medicine & Dentistry, London (ON), Canada

**Keywords:** medical education, continuing professional development, role reversal, phenomenography

## Abstract

**Background:**

Medical education traditionally involves directional flow of knowledge/skills/attitudes from a senior to junior individual. However, medical training also provides opportunities for expertise role-reversal, where the direction of flow is reversed. Unlike fields such as aviation, medicine has not yet begun to fully realise the educational potential of this approach.

**Objective:**

To better understand how role-reversal is viewed by medical education participants, necessary for its use as a tool to advance both education and patient care.

**Methods:**

A senior resident designed and led a feedback-writing workshop for her own consultants (conducted 2022). After the session, eight consultants were interviewed in a semi-structured format. Analysis was conducted using the Stenfors-Hayes phenomenographical approach.

**Results:**

A multiplicity of experiential perspectives was identified by both consultants (teacher/participant/supporter/hierarchy member/colleague/holder of multiple perspectives) and trainee (presenter/subordinate/learner/researcher). The exercise increased appreciation and awareness of the complexity of the trainee-consultant educational-power relationship, though both parties maintained traditional hierarchy despite altered informational flow. Participants often held multiple articulated experiential perspectives simultaneously.

**Conclusions:**

Consultants were able to assume a learning mindset while simultaneously maintaining awareness of their existing hierarchical relationship to the trainee-presenter; the trainee, conversely, struggled to adopt the teacher mindset. Deliberately viewing moments where trainees present new information to consultants as expertise role-reversal may provide a starting point for more equitable knowledge exchange between both parties in the clinical routine, and a foil for epistemic injustice. Increasing recognition and use of expertise role reversal can play a critical role in improving educational culture.

## Zusammenfassung

**Hintergrund:**

Während der ärztlichen Ausbildung werden traditionellerweise Wissen und Fähigkeiten von erfahrenen Ärzt:innen und Fachkräften auf die auszubildenden Ärzt:innen übertragen. Die Ausbildung bietet jedoch auch Gelegenheiten zur didaktischen Rollenumkehr, wo ältere oder erfahrenere Ärzt:innen von jüngeren lernen. Im Gegensatz z.B. zur Luftfahrt, ist dieser Ansatz in der Medizin in seinem didaktischen Potential noch nicht gründlich erforscht.

**Zielsetzung:**

Die Zielsetzung dieser Studie ist es, zu untersuchen, wie Teilnehmende an einer ärztlichen Fortbildung eine didaktische Rollenumkehr erleben. Dies könnte sowohl in der Ausbildung als auch in der Praxis zu Verbesserungen der Zusammenarbeit und des Wissensaustauschs führen.

**Projektbeschreibung:**

Ein:e Ärzt:in im 2. Jahr ihrer Facharztausbildung (Onkologie, „resident“ im kanadischen Ausbildungssystem) führte eine an ihre Ausbilder („consultants“) gerichtete Fortbildung durch. Das Thema der Fortbildung war das Verfassen von schriftlichem Feedback. Acht an der Fortbildung teilnehmende Fachärzt:innen und die vortragende Ärzt:in nahmen danach an halbstrukturierten Interviews teil; die Daten wurden phenomenografisch nach Stenfor-Hayes ausgewertet.

**Ergebnisse:**

Es konnten mehrere Perspektiven identifiziert werden, auf deren Basis die anwesenden Fachärzt:innen und die auszubildende Ärzt:in die Fortbildung bewerteten. Dazu gehörten: Lehrkraft, Workshop-Teilnehmer:in, Unterstützer:in, Kolleg:in, Vorgesetze:r innerhalb der medizinischen Hierarchie (Fachärzt:innen), bzw Vortragende, Untergebene, Forscher:in (vortragende Assistenzärzt:in). Teilnehmende beschrieben auch mehrere gleichzeitig eingenommene Perspektiven. Die Fortbildung führte allseits zu einer klaren Anerkennung und tieferem Verständnis des Arbeitsverhältnisses und der Ausbildungsverantwortung zwischen Lehrkräften und Auszubildenden. Die deutlich unterschiedlichen Positionen in der traditionellen Hierarchie wurden jedoch durchweg gefühlt eingehalten, trotz der umgekehrten Richtung der didaktischen Tätigkeit.

**Schlussfolgerung:**

Fachärzt:innen und auch der/die anwesende Chefärzt:in konnten sich in die Rolle der/des Lernenden hineinversetzen, obwohl sie sich gleichzeitig der Verantwortung und Weisungsbefugtheit gegenüber der auszubildenden Ärzt:in bewusst waren. Diese jedoch tat sich zunächst schwer damit, innerlich die Rolle der Lehrkraft einzunehmen. Im klinischen Alltag gibt es häufig Situationen, in denen Assistenzärzt:innen, PJler, usw Fachärzt:innen oder Chefärzt:innen neue oder zusätzlich Informationen zu ihren Patienten geben, und solche Situationen könnten auch als didaktische Rollenumkehr aufgefasst werden. Falls eine situationsbezogen bewusste didaktische Rollenumkehr zu einer akzeptierten Praxis während der ärztliche Ausbildung werden sollte, könnte dies auch der epistemischen Ungerechtigkeit entgegenwirken, die sonst leicht aufgrund der Differenzen innerhalb der medizinischen Hierarchie auftreten kann und etwaige wichtige Beiträge und Einblicke innerhalb eines Teams unterbinden kann.

## 1. Introduction

Written feedback in medical education ideally provides essential instructions for growth, as well as formalising a record of learning. However, the feedback often varies in quality [1], which subsequently influences its utility in enhancing a learner’s performance/skillset. There have been numerous interventions documented in the literature aimed at improving the quality of written feedback; however, these have been lukewarm in their successes, with a number of limitations, ranging from feasibility and buy-in to tangible improvement [2], [3]. 

Despite longstanding calls for feedback to be dialogic and conversational [4], [5], it typically continues to involve a directional flow of knowledge, skills, or attitudes from a senior to a junior individual. However, scenarios in medicine where the traditional directional flow is modified have been established. Such situations include co-learning, where trainees and consultants learn together to address a mutual knowledge gap [6], or reverse mentoring, where a more junior mentee provides new, fresh insights and guidance to an older mentor [7]. Both of these models highlight alternatives to the traditional directional flow of medical education, providing options for informational flow and, consequently, knowledge acquisition for all members of the team, which could benefit team members’ own learning as well as task accomplishment. Conversely, instances of so-termed “epistemic injustice”, have also been well-documented in the literature [8]. Epistemic injustice is a negative psychological reaction to unrecognised expertise role-reversal and may be triggered by a devaluation or failure to acknowledge a more junior individual’s contributions or knowledge. Epistemic injustice and its sequelae, including moral distress and weakening of the trainee-consultant relationship, are acknowledged in various healthcare professionals, but have serious implications when considered in the trainee (future healthcare professional) populations, including decreased retention in the field, increased burnout, and decreased job satisfaction [8], [9], [10], [11]. 

Instances of complete reversal of insights, guidance, and/or knowledge (from junior to senior) are more commonly embraced in fields outside medicine. In aviation, junior individuals freely provide suggestions and advice to more senior individuals [12]. This practice forms an essential part of “crew resource management”, a form of emergency management that consciously rejects hierarchical norms and utilizes all available expertise [13]. While this may be seen as a focus on psychological safety (the learner feels safe speaking up) [14], as well as expertise role-reversal, the contribution of the junior individual has to be deliberately recognised for successful knowledge transfer. This allows for innovation and teamwork aimed towards optimal outcomes in unusual or high-risk/high-stress situations. As another profession where decisions may have severe consequences and, therefore, clear-eyed decision-making is necessary, medical education can learn much from aviation in how to facilitate knowledge sharing [12]. Indeed, enhancing teamwork through deliberate training of learners’ speaking up skills, and supervisors’ own feedback receptivity, is an ongoing educational strategy [15], [16].

The improvement of written feedback provides a natural opportunity to consider utilising an expertise role-reversal approach in medicine. As direct recipients and beneficiaries of faculty feedback, trainees are in an ideal position to coach faculty members on ways to improve written feedback. One method previously trialled in higher education to address the issues surrounding subpar feedback is a reversed-role faculty development session, during which learners provide instruction to faculty [17]. This is a strategy we seek to explore further within medical education, where individuals in traditional “student” roles lead faculty development sessions instructing their teachers on how to provide optimal feedback. 

It is essential to acknowledge that hierarchies inherent to medicine (and the hidden curriculum) might cause some trainees to balk at the prospect of teaching their superiors, or cause some faculty to distrust an educational presentation by their subordinates. Exploring these feelings is essential to understand the feasibility of expertise role-reversal scenarios and identify potential barriers to broader implementation.

Consequently, we conducted a resident-led feedback writing workshop grounded in expertise-role reversal, with the goal to understand how the session was experienced by participants. 

## 2. Methods

Given our goal of exploring the social experience of a role-reversed educational activity, a qualitative research approach was chosen. Within a constructivist epistemology, phenomenography was chosen as the specific methodology. Phenomenography refers to a research approach in which small snippets of described experience are used to build a holistic picture of a situation [18], including individual variations in the experienced situation, from the perspective of those experiencing the situation [19]. This was an appealing methodology as we were focused on understanding the possible broad landscape of faculty members’ responses and lived experiences of the phenomenon of a trainee-led workshop. The research paradigm involved a relativist ontology (reality is based on individual experiences), constructivist epistemology (knowledge of reality is constructed by individuals), and constructivist paradigm (individuals construct reality through their perceptions of the world around them). 

**Figure 1:**
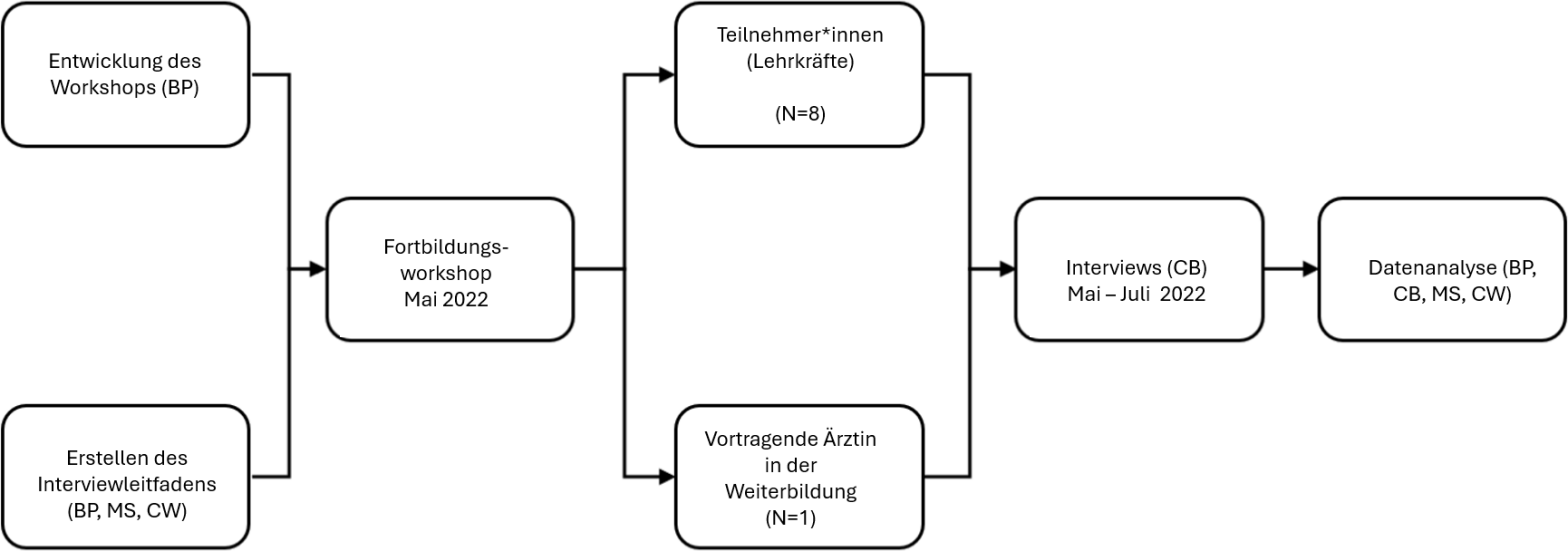
Timeline

The faculty development session itself consisted of a single-session, in-person workshop aimed at medical oncologists in a tertiary-care academic centre in Ontario. The session was designed and led in 2022 by a then-fifth-year medical oncology trainee (BP) at the same centre as faculty members MS and CJW, and based on Gagne’s model of instructional design [20]. The workshop lasted two hours; the first half was didactic with interactive built-in questions, and the second half involved simulated feedback scenarios with a standardised student. Oncologists were recruited in advance of the session for participation in the study; workshop participation without study participation was also permitted. Shortly after the session, consenting faculty members (n=8) participated in a short, semi-structured, face-to-face interview (see figure 1 (Fig. 1)). Interviews were conducted by a dedicated then-non-oncologist, non-workshop-participant physician (CB) who received training in qualitative interviewing, and then recorded and transcribed using transcription software, with a manual check (see table 1 (Tab. 1)). One participating faculty member (MS) was a member of the study team. An interview was also conducted by CB with the trainee presenter using the same approach. 

Analysis was conducted in accordance with the framework laid out by Stenfors-Hayes [21]. Following transcription, inductive coding was performed; codes were then grouped into categories to explain the experience of expertise role-reversal in the context of a trainee-led workshop. A member check was performed after initial coding by sending a copy to participants to ensure their perspectives had been accurately captured. An audit trail was also kept by the first author to assist with study confirmability and dependability [22]. Analyses were reviewed and agreed upon by all team members.

This study received ethics approval from the western research ethics board.

## 3. Results

### 3.1. Consultant roles

Seven experiential perspectives were identified by consultant participants during the workshop. These highlighted the ability of consultants to place themselves in the role of a learner when they felt this mindset might be of benefit, yet simultaneously maintain other roles, some of which were more natural or typical for the consultant. The seven experiential perspectives identified by participants were: 

#### 3.1.1. Perspective #1: Consultant as a teacher/mentor/coach of trainees in general

The session was seen as a good opportunity for any trainee, both to practice presenting and to demonstrate knowledge in the content area. Consultant attendees were also interested in the session topic and appreciated learning about ways to improve their teaching skills. They saw room for improvement in their own feedback practices (most were highly motivated to attend session based on content alone). 

*We’re always talking…about teaching. But I never know what, in fact, the trainee or the medical student or the rest of the fellow[s] really wants to get from us. So I think having specifically [Trainee] talking about those things in that workshop was quite helpful.* (Participant 2)

*We need to give feedback so often and generally .., with very little time on our hands to do it properly. That is always quite challenging. And so… I was really kind of appreciative that actually [trainee] had taken on a very challenging part of assessment, which is providing feedback. So I was looking forward to it.* (Participant 4)

#### 3.1.2. Perspective #2: Consultant as a participant in a faculty development session

Generally positive feedback towards session content and delivery was conveyed, with particular emphasis on the trainee’s presentation style and skill. Some feedback was constructive, such as lack of humour during the presentation, or a sense of distraction from exaggerated demonstrations. The workshop itself was positively received and seen as effective, and specific learning points were remembered and discussed. The trainee presenter, as a senior trainee and a trainee in the consultants’ own division, was seen as particularly credible, which also contributed to session buy-in. Pre-existing knowledge of and relationship with the trainee, and knowledge of trainee’s interests and training in education, also influenced the experience by adding further interest in/buy-in to the session. More broadly speaking, participants voiced enthusiasm at attending another trainee-led session in the future, but some were conflicted on whether trainees could teach scientific or medical content (whether alone or with consultant guidance), as consultants are the content experts in these fields. Consultants also commented on the trainee’s credibility, citing prior knowledge of the trainee which increased credibility, as well as conveying scepticism about a session led by a trainee with less credibility.

*I think like having a trainee like [trainee] do that, knowing her, knowing how she interacts with us, knowing her skills. If there was a different trainee that I didn’t know, I would probably be a bit more skeptical to go.* (Participant 2)

*It probably made it different than if, say, a regular PGY-5 who maybe has not shown an interest in education or something, said, I want to talk to you guys about feedback. That would be that would be a different thing for me. I mean, it would be interesting, but I don’t know if I would have the same confidence that they would be able to bring something to me that I could learn from.* (Participant 3) 

#### 3.1.3. Perspective #3: Consultant as an empathetic supporter of the trainee

Participants reported concern for the specific trainee and for generic trainees in positions of teaching their own consultants. Consultant support for a trainee leading a session was felt to be mandatory, although no consultants were involved in session preparation or execution. Simultaneously, it was noted by participants that the trainee was receiving tacit support from a mentor in the audience during the presentation. Concern for the specific trainee was noted by quite a few participants, citing presumption or knowledge of the trainee’s anxiety or intimidation leading the session. Simultaneously, however, consultants were pleased for the trainee to have opportunity to present, and to have done so well. 

*That was the one thing I was…a little worried, you know, for a trainee…providing that kind of a session can be a little intimidating because, you know, it's among the staff that they may have worked with already.* (Participant 4)

*I think just knowing her personally shaped my expectations of what I was going to get and my enthusiasm to attend it and engage with it, because obviously she's one of our residents. I kind of also have this feeling that you want to support her and what she's passionate about in education.* (Participant 7)

#### 3.1.4. Perspective #4: Consultant as a researcher

Participants noted reflecting on the session, both during the session and afterwards, in anticipation of interview. They also noted that comfort/effectiveness of trainee-led workshop would depend on the specific trainee, consultants, and setting; not every trainee can present every topic to every group (again relating to trainee credibility). Comparisons normalising the experience were made to patient-reported data, and that a trainee presenting trainee experience or a medical education topic is similar to a patient presenting patient experience. This, again, serves as a foil to a trainee presenting in an area where a consultant is seen as an expert. 

*You look at clinical trials, patient reported outcomes, which by the way, I think they’re done [poorly]. They’re still being done from the point of view of investigators and truly like not what matters to patients. And, you know, so all of this issue of outcomes…when you’re actually able to have time with the end users, like the people that are the ones experiencing what’s going on, that's the most valuable information that you can have. Right? So especially when it's around something like this where it’s feedback.* (Participant 6)

#### 3.1.5. Perspective #5: Consultant as a member of an existing hierarchical structure/assessor of trainee

Several consultants described seeing the senior trainee as closer to a colleague than a trainee, which impacted their experience of the session as “trainee-led”. Indeed, the opinion of workshop seems largely shaped by the opinion of the trainee, and the perceived credibility of the trainee seemed to play largely into the workshop experience. 

*So my expectations were pretty high um, and I knew how much she cared about this. So I knew she was probably going to have put a lot of thought and effort into it. So I actually had very high expectations of her*. (Participant 6)

Participants did note that some consultants might take umbrage to being taught by a trainee, and some expressed skepticism at being taught by more junior or less credible learners. Indeed, credibility of the trainee presenter was discussed by many participants, with factors such as formal training in education, interest in the field, and personal knowledge of the trainee’s skills lending credibility. It was recognised that the direction of informational flow was reversed during the workshop, but consultants felt that the hierarchy was still maintained. The session was viewed as a performance by a trainee towards consultants, similar to a case presentation in a clinical session. Awareness of a traditional culture of power or hierarchy between consultants and learners was also mentioned. Some consultants proposed breaking down the hierarchy for freer exchange of ideas/information between consultants and trainees. In general, however, consultants highlighted an increased appreciation of the complexity of the trainee-consultant educational and power relationship after the workshop.

*Until our culture changes, until like the power shift, the hierarchy…the fact that, you know, faculty are the ones that are filling out the evaluations… [faculty are] the ones that are part of the process of determining whether people progress through…that is really still heavily prevalent in this culture, right. So until that changes I think [awareness of hierarchy] won’t change*. (Participant 6)

#### 3.1.6. Perspective #6: Consultant as a colleague of other audience members

Pre-existing collegial relationships influenced interpretation of room’s atmosphere and general awareness of the room. For example, interruptions from colleagues were appreciated by consultants who were hoping for humour or distraction during drier parts of the workshop. 

*I know some of my colleagues, how they like to interact. I know that this kind of didactic thing would not be probably received [well]…So that’s why I got a little bit uncomfortable there. About how long is that going to last before someone speaks up?* (Participant 8)

It was also noted that a multitude of perspectives might work best to engage the faculty in the room:

*I don’t think you should be [doing] only trainee workshop. It should have a faculty as well. The same applies for workshops only with faculty; may not work as well. You have to have a combination of all the levels of trainees and faculty … because that combination of experience with appetite to learn is important.* (Participant 2)

#### 3.1.7. Perspective #7: Consultant as a holder of multiple articulated perspectives

Consultants reported feeling conflicted during the workshop and becoming aware of possessing multiple, simultaneously-articulated perspectives, such as empathising with the trainee presenter while being interested in session content, or being anxious/guilty on behalf of the trainee and proud of the trainee. Consultants described putting themselves in the trainee’s shoes, switching in-and-out of a teacher or participant role and more into an empathetic supporter role. A dichotomy was explicitly described between the consultant as a learner and as an assessor of the trainee during the session. Consultants described switching between roles deliberately to handle moments of vulnerability, as well as occasionally feeling unsettled when trying to reconcile conflicting, equally-significant roles.

*Well, I was…very aware of my own ideas. So obviously immediately the reaction came to me primarily, as I’ll say, as a presenter side, oh no someone’s speaking up. But then I sort of recognized that and said, no, I’m going to just stay neutral and see how this plays out and suppress my own desire for this colleague of mine to you know, well, to shut up to put it in slang terms. And it turned out actually to be quite, quite good, because the other side of me, the audience member, uh, sort of wanted that, that bit of a break from the one directionality of it and welcomed the dialogue and the relaxation of the atmosphere a bit. *(Participant 7).

### 3.2. Trainee roles

Four experiential perspectives were identified by the trainee leading the workshop. These were: 

#### 3.2.1. Perspective #1: Trainee as a presenter/teacher

The trainee engaged in extensive, intense preparation for the session, representing in retrospect a response to her discomfort with teaching her own consultants. Session design and preparation were constructed, in part, to maintain control of the room; a didactic teaching style and highly-structured mode of audience interaction were anticipated to help project authority and facilitate the reversed information flow. Maintaining control of the room’s dynamic and presentation structure was used to mitigate fear, anxiety, and insecurity around teaching the trainee’s own consultants. Reflection revealed that both the trainee’s preparation intensity and her own negativity towards interactivity/spontaneity was driven by anxiety and discomfort. 

*I think the didactic portion was probably the most intense (ly prepared) just because I knew that would be me, and I want to get across what I wanted to say in the time frame I had to say it. In terms of the more interactive second part with the… the workshop itself, I certainly prepared a lot less…so the prep work there was actually a bit less just because it was something I was more comfortable with.* (Trainee)

#### 3.2.2. Perspective #2: Trainee as a subordinate in a medical hierarchy

The trainee voiced repeated anxieties and insecurities related to teaching her own consultants; respected consultants were described as ultimate authority figures, and she voiced concerns about meeting expectations, despite the novel nature (and consequent lack of expectations) of the teaching structure. She also cited concerns about perception of credibility, and deliberately selected a workshop topic and angle to maximise credibility, as well as designing the talk to reflect the opinions of many trainees (not just hers, which was emphasised multiple times). The trainee reflected on expecting negative response to the workshop, and was surprised by positive responses and feedback to the workshop, which was seen as validating and supportive by those with more experience/in higher positions. 

*So the fact it was faculty development and there were faculty members that I knew. And who I would have an ongoing relationship with and who I felt, some of whom may be judging me quite harshly, um certainly made me feel that I had to… pull my socks up with this one. Versus this teaching session I led today, it was with a group of new residents. I feel like the judgment there is going to be quite a bit less, and it’s also something I feel more comfortable in versus teaching a group of my consultants. *(Trainee)

#### 3.2.3. Perspective #3: Trainee as a learner in a teaching programme

The trainee was in a performance mindset during the preparation for and execution of the workshop, and, again, voiced repeated concerns about fulfilling consultant expectations. It was also clear that the trainee had pre-existing relationships with and perspectives of different audience members, which also influenced both anxieties and development/presentation of the workshop. 

*I guess really what was bothering me more than anything else was just the thought of messing up or screwing up and looking like a big fool in front of everybody…uh, which as a PGY 5 who is you know looking at fellowships and jobs and where reputation is very important and kind of one of the few currencies that you have because, you know you don’t come with big grant money, you don’t have all these publications to your name you're kind of a nobody, and the only thing that you have is potential and a reputation. And to put yourself in this situation… is to make yourself quite vulnerable.* (Trainee)

#### 3.2.4. Perspective #4: Trainee as a researcher 

The trainee also had awareness of multiple experiences in the moment. For example, the trainee reflected on duality as a researcher answering a question versus an individual delivering a judged performance to her own consultants. 

*And um… I kind of have to teach a good session, otherwise the issue is definitely with me and not with this format of teaching, so trying to really bring my A game, um, and to avoid letting people down.* (Trainee)

### 3.3. Participant learning

Although not the primary focus of this study, it should be noted that faculty recalled several salient points they had learned, suggesting the session was effective. During the interviews, consultants were able to recall key learning points which had influenced their feedback writing practice, including several feedback structures discussed, deliberate word choice, and focusing on the trainee’s mindset and feedback receptivity in the moment. They appreciated the trainee perspective around written feedback, because some more senior faculty had not personally experienced receiving the type of constructive feedback they were now expected to provide.

*“This is a different world for all of us because most of us have not been the resident who's getting feedback on an observed encounter. And so what someone wants to hear or not hear in that kind of feedback ---- We can't put ourselves in that person's shoes because we haven't lived that experience. So therefore, a faculty member talking about it is not the same as someone who actually has kind of grown up in this different type of CBD [Competence by Design] type learning”.* (Participant 3)

## 4. Discussion

In summary, this study explored the experience of participants in a workshop using expertise role-reversal to teach feedback writing. Key takeaways include the positive overall reception and perceived efficacy of the workshop by consultant participants, who were highly receptive to being taught by a trainee.

Given the established hierarchies, expectations, and cultural norms inherent to medicine [23], there was a priori concern that these conventions might prevent learning in an expertise role-reversal setting by acting as immutable and unarticulated aspects of the hidden curriculum. However, the success of this study and the participants’ reflections emphasise that expertise role-reversal is recognisable (and embraceable) by consultants. Faculty could have had concerns about being taught specifically around a core teaching skill (namely, giving feedback) by a more junior person, as this might have threatened their professional identity as an educator; however, this was embraced and even compared to patients reporting on medical topics at conferences and seminars. Indeed, there were several references and metaphors comparing learner-led faculty development with patient involvement in care and physician education. The comparison of trainees to patients can potentially be seen as playing into existing hierarchies, where consultants can obtain information and feedback on their practices, but still remain largely in their hierarchal positions. However, analogous to increased patient autonomy and input in shared decision-making, a bidirectional, dialogue-based interaction between consultant and trainee may move education forward towards a new paradigm. In shared decision-making, physicians contribute experience around treatments and patients bring their perspectives around what matters to them [24]. Our study suggests that in the educational context, a similar sharing of educational roles may be possible. Senior physicians contribute experience around skills development, and junior physicians or trainees contribute fresh perspectives which can inform how the skills development is enacted. However, achieving this sort of dialogue requires courage and openness. Along these lines, although the trainee did report substantial anxieties and imagined negative repercussions around the workshop, she was still able to work through the discomfort of performing outside a conventional trainee-consultant relationship to deliver a well-received workshop. In other words, the trainee did perceive risk to the expertise-role reversal, but was still able to participate. However, weight of the medical hierarchy and medical culture was a strong theme in the trainee’s reflections, as well as consultant interviews. 

As a single-department, single centre study, our results are strongly influenced by the existing culture in the department, which does serve as a limitation of the study. The openness of consultants towards learning from their trainees will likely be influenced strongly by existing traditions, which will vary from one department to the next, even within the same institution. Similarly, trainees will vary widely in their experienced psychological safety when interacting with the same group of consultants, depending on their own developing professional and personal identities, as well as personality, prior experiences, and dynamics in the moment. Nonetheless, elements of the experiences of both trainees and consultants in our study would possibly be found to varying degrees if the study were to be repeated with other individuals. Further research confirming the essential experience domains we identified is encouraged. 

Based on the positive reception of and feedback for the trainee-led faculty development session, we hope that this mode of teaching might be used and explored further, especially in the context of medical education faculty development. As end-users of medical education, trainees are in an ideal place to provide education to faculty members regarding educational practices. 

## 5. Conclusion

In summary, we have presented a feedback-writing workshop designed and led by a trainee; this was well received by consultants, who were able to assume a learning mindset while simultaneously being aware of their existing hierarchical relationship to the presenting trainee. It was more difficult for the trainee to overcome concerns related to pre-existing fears and anxieties and to view herself primarily as a teacher, suggesting that such concerns must be deliberately addressed as well, in order for a bidirectional information flow to occur in clinical settings. However, deliberately viewing moments where a trainee is presenting information that is not known (and can’t be known) by the consultant at that point in time may serve as a starting point for more equitable exchange of knowledge between trainees and consultants in the clinical routine 

## Authors’ ORCIDs

- Beatrice Preti: [0000-0002-3664-417X]
- Michael Sanatani: [0000-0002-2423-7171]
- Christopher Watling: [0000-0002-1440-2401]

## Competing interests

The authors declare that they have no competing interests. 

## Figures and Tables

**Table 1:**
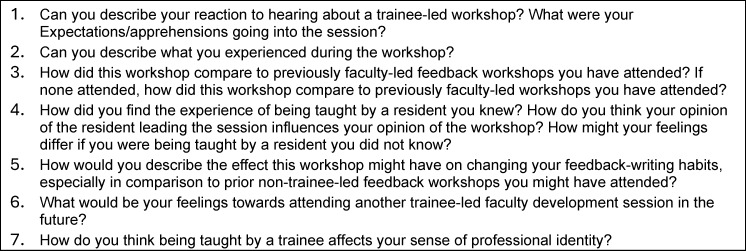
Faculty semi-structured interview prompt questions
